# The use of *Galleria mellonella* to study microbial interactions, immune responses, and substrate interactions

**DOI:** 10.1042/EBC20250030

**Published:** 2026-09-02

**Authors:** Evgenia Maslova, Ciaram Staber, Ronan R. McCarthy

**Affiliations:** 1 Division of Biosciences, Department of Life Sciences, College of Health and Life Sciences, Brunel University London, Uxbridge UB8 3PH, U.K.; 2 National Biofilms Innovation Centre, School of Biological Sciences, University of Southampton, Southampton SO17 1BJ, U.K.

**Keywords:** bacterial interactions, biofilms, commensals, Galleria mellonella, infection, wound healing

## Abstract

Interbacterial interactions are diverse and complex and often dependent on the given niche these bacteria inhabit; therefore, it is often necessary to validate *in vitro* findings *in vivo*. Multiple mammalian *in vivo* models have been established for this purpose, from mice to larger vertebrates like pigs. However, the use of mammalian models comes with high costs and strict ethical considerations, which can limit cohort sizes and experimental outputs. As a result, there has been a significant rise in the use of the *Galleria mellonella* (greater wax moth larva) research model, not only as a model for drug toxicity and virulence determination but also as a model for studying more complex bacterial and interkingdom interactions *in vivo*. The versatility of this model and the capacity to carry out high-throughput experiments make this a particularly attractive platform to study bacterial community dynamics. The use of this invertebrate research model organism to study bacterial interactions alleviates the bottleneck between *in vitro* and *in vivo* research while also contributing to the replacement and reduction of higher animals needed for *in vivo* studies. In the present review, we discuss the use of *G. mellonella* to investigate microbial interactions *in vivo*, including studies that investigate interbacterial relationships, interkingdom interactions, the host immune response, and the role of bacteria-substrate interactions.

## Introduction

*Galleria mellonella*, the greater wax moth larva, has gained popularity in recent decades amongst the scientific community. It has become established as a robust *in vivo* research model in multiple research fields, from infectious diseases to ecotoxicology [1,2]. *G. mellonella* have been used to study microbial interactions *in vivo*, including interbacterial interactions, host–pathogen interactions, immune responses towards bacteria, and bacterial interactions with various substrates. While morphologically distinct from more traditional mammalian model organisms, *G. mellonella* possesses several specific features that make it an attractive *in vivo* model. They possess a thin cuticle, which acts as a protective barrier. The cuticle is made up of chitin, proteins, lipids, phenols, and chinons [3–6]. The larvae have an open circulatory system (haemocoel), which means that the haemolymph (similar to blood in mammals) is free-floating around the larval body [7]. They also possess tissue ferritin and protein-bound iron, which have been shown to be essential to the immune function of the insects [8–10]. The predominant carbohydrate in larval haemolymph is trehalose [11]. *G. mellonella* have no adaptive immune system, but they do possess a robust innate immune system that comprises of immune cells similar to mammalian macrophages and neutrophils, and various humoral factors such as pattern recognition receptors, antimicrobial peptides (AMPs), and opsonins [12]. Wound healing in *G. mellonella* occurs through re-epithelisation, a process that is similar to wound healing in humans, enhancing the relevance of this model to study infections and evaluate novel therapeutic interventions [13]. While the model has developed, so too have the breadth of experimental outputs and associated protocols. For example, it is now possible for researchers to track infection progression in real time through enumerating the haemolymph bacterial bioburden, digitally monitoring larval melanisation and movements, and measuring host transcriptional responses. When comparing *G. mellonella* to other animal models, the major differences and limitations of this model are the lack of adaptive immunity, an open blood circulation system, and the general relative anatomical simplicity in comparison to more complex models. Despite these limitations, it also overcomes several barriers associated with mammalian model organisms, such as affordability, maintenance, cohort size, and ethical challenges. Furthermore, *G. mellonella* models provide rapid experimental outputs and enable high-throughput studies, increasing the statistical robustness and reliability of the data through the use of larger experimental cohorts.

In the present review, we discuss the use of *G. mellonella* to study bacterial interactions, covering interbacterial interactions, host–pathogen interactions, and interactions between bacteria and various substrates.

## Interbacterial interactions

The nature of interbacterial interactions is complex, diverse, and often specific to a given ecological niche. This includes competitive, cooperative, and antagonistic relationships within the bacterial community as well as neutral coexistence [14]. While the evaluation of these interactions *in vitro* is useful, there is an increasing appreciation that many of the reported *in vitro* interactions need to be validated *in vivo*. *In vivo* validations of findings related to therapeutic discoveries and pharmaceuticals are an essential stage in the drug development pipeline and are typically required by regulatory bodies. The use of *G. mellonella* models provides a high-throughput replacement model for researchers to rapidly evaluate their *in vitro* findings in a living organism without the financial and logistical hurdles of securing a higher mammalian model [15].

Exploring microbial interactions in classic laboratory *in vivo* models such as mice, rats, or larger animal models such as pigs is often constrained by strict ethical approval requirements, while experimental scope is further limited by regulatory restrictions on cohort sizes and the high costs associated with large-animal studies. The *G. mellonella* model offers a unique opportunity for investigating these interactions *in vivo* due to several biological, practical, and ethical factors. This includes the capacity to be infected by a range of human pathogens and commensals and for experiments to be carried out at 37°C. From a practical and ethical standpoint, the exclusion of *G. mellonella* from the Animal Scientific Procedures Act 1986 (U.K.) means they can be utilised in high-throughput studies with a wide range of host/pathogen biological outputs, making them an attractive and low-cost model to study microbial interactions *in vivo*. The majority of studies have utilised the haemocoel injection model to inoculate the larvae with organisms of interest, although the recently developed burn wound model has made it possible to study topical interactions also [16–18].

**Figure 1:**
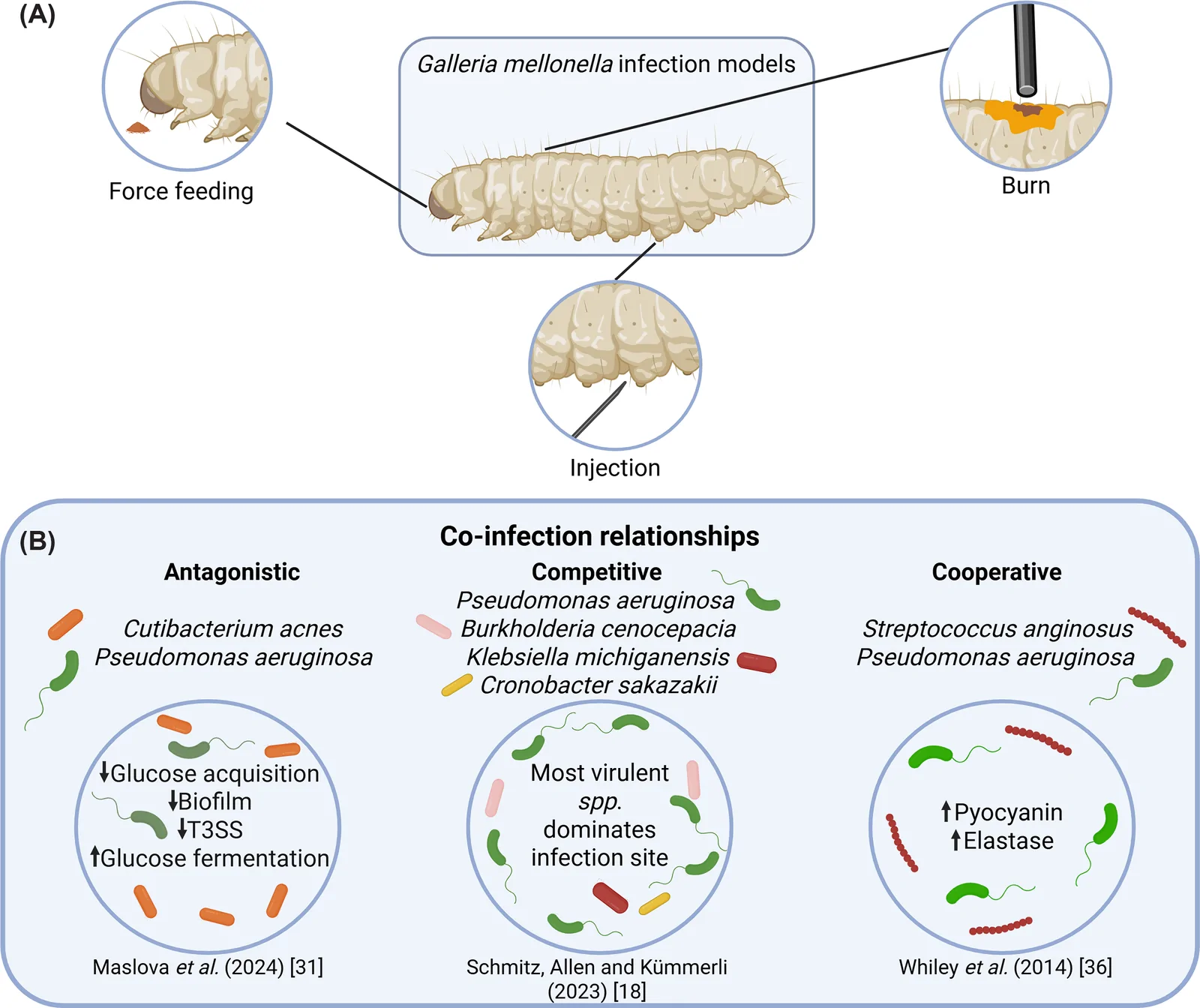
Schematic representation of *G. mellonella* applications for investigating interbacterial interactions The upper half (**A**) of the figure depicts experimental methods in which bacteria are introduced to *G. mellonella* larvae. These include force-feeding, injection, and burn models. The lower half (**B**) shows example interactions described in the literature within the *G. mellonella* model, including antagonistic, competitive, and cooperative co-infection outcomes. This figure was created based on references [18,31,36] and was generated using BioRender.com.

The *G. mellonella* co-infection model has been extensively used to characterise the relationship between several opportunistic human pathogens (Table 1). In one study, *Pseudomonas aeruginosa, Burkholderia cenocepacia, Klebsiella michiganensis*, and *Cronobacter sakazakii* were chosen to investigate interspecies dynamics within a polymicrobial infection context. These species were selected due to their high co-occurrence rate in human infections. *P. aeruginosa* and *B. cenocepacia* are commonly co-isolated from lung infections, and *P. aeruginosa* and *K. michiganensis* are commonly co-associated with burn wound infections [19]. Firstly, they characterised how lethal each bacterium was on its own in the *G. mellonella* utilising the haemocoel injection model. They also showcased the impact that larval age and infection dose have on larval mortality. With these findings, they standardised the infection dose to 10^5^ CFU for mixed bacterial infection testing. This fixed amount of CFU was also verified to create a hierarchy of virulence of each bacterium as an infection agent in the larvae, allowing them to be ranked individually [19]. The results showed that in the mixed infection scenarios mortality of the host is often dependent on the pathogenicity of the most virulent strain in the cohort (Figure 1). Monitoring bacterial bioburden in the haemolymph suggests that the more virulent strains in mixed infections also tend to be the most abundant [19]. Furthermore, it demonstrated that targeting the most virulent species in a mixed species infection significantly increases *G. mellonella* survival [19]. Additionally, the relationship between *P. aeruginosa* and *Staphylococcus aureus* clinical isolates has been characterised by Gomes-Fernandes *et al.* [20]. The present study revealed that co-infection produced an additive effect in mortality in the *G. mellonella*, which is contrary to the *in vitro* studies that demonstrated a competitive relationship between *P. aeruginosa* and *S. aureus*, where *P. aeruginosa* produced inhibitory factors such as proteases, rhamnolipids, and pyocyanin to limit *S. aureus* growth [20]. However, the present study did not confirm the effect of *P. aeruginosa* and *S. aureus* co-infection on bacterial bioburden from *G. mellonella* haemolymph, which could provide greater insight into the relationship between these bacteria *in vivo*.

**Table 1:** Interbacterial interactions reported in the *G. mellonella* model

Bacteria	Interactions with	Interactions *in vivo*	Reference
*P. aeruginosa*	*S. aureus*	Synergistic for some co-isolated *S. aureus* + *P. aeruginosa* combinations	[20]
	*S. anginosus*	Synergistic in both	[36,37]
	*B. cenocepacia*	Competitive	[19]
	*K. michiganensis*	Competitive	[19]
	*C. sakazakii*	Competitive	[19]
	*C. albicans*	Additive when primed and infected by the same pathogen.	[25]
	*B. thuringiensis*	Additive when primed and infected by the same pathogen.	[25]
	*P. entomophilia*	Additive when primed and infected by the same pathogen. Priming with *P. entomophilia* enhances infection with *B. thuringiensis*.	[25]
	*Lactobacillus spp*	Inhibitory with co-inoculation. Inhibitory with cell-free supernatant.	[29–31]
	*C. acnes*	Inhibitory with co-inoculation	[32]
	*E. coli*	Inhibitory	[28]
*C. albicans*	*S. aureus*	Synergistic	[21]
	*G. vaginalis*	Synergistic	[22]
	*P. brasiliensis*	Synergistic	[18]
	*P. aeruginosa*	Additive when primed and infected by the same pathogen.	[25]
	*B. thuringiensis*	Additive when primed and infected by the same pathogen.	[25]
*A.baumanii*	*K. pneumoniae*	Synthropic	[17]
*A.fumigatus*	*P. aeruginosa*	Synergistic	[23]
		Antagonistic	[24]
*Lactobacillus spp*	*E. coli*	Antagonistic	[26]
	*N. gonorrhoeae*	No interaction	[27]
	*L. monocytogenes*	Antagonistic	[34]
	*E. faecalis*	Antagonistic	[35]
*E. coli*	*S. typhimurium*	Antagonistic	[33]

The *G. mellonella* model has been used to characterise the relationships between not only bacterial pathogens but also interkingdom relationships between fungal pathogens and bacteria (Table 1). An example of this is the finding that *Candida albicans* augments *S. aureus* pathogenicity in co-infection assays. Similar virulence-potentiating effects were observed in co-infections with *C. albicans* and *Gardnerella vaginalis* and *C. albicans* and *Paracoccidioides brasiliensis* [18,21,22]. The *G. mellonella* co-infection model has also been extensively used to investigate the relationship between *Aspergillus fumigatus* and *P. aeruginosa*, two pathogens that commonly co-infect the lungs of patients with cystic fibrosis [23,24]. Reece *et al*. showcased that pre-exposure of *G. mellonella* to non-lethal concentrations of *A. fumigatus* increases the mortality from the consequent infection with *P. aeruginosa*. Scott *et al*. highlights the importance of *P. aeruginosa* volatile sulfur compounds and argues that they can enhance the infection by *A. fumigatus*. *G. mellonella* has also been used to study the relationship between *Pseudomonas entomophilia, Bacillus thuringiensis, C. albicans*, and *P. aeruginosa* by inoculating the larvae with *P. entomophilia, B. thuringiensis*, and *C. albicans* prior to the infection with *P. aeruginosa*. The present study revealed that the larvae that were pre-exposed to bacteria were only more resistant to lethal bacterial CFU doses of the same pathogen. Moreover, in the case of pre-infection with *P. entomophilia*, the larvae were more susceptible to further infection with *B. thuringiensis* [25].

Bacterial interactions involving *Lactobacillus* species have been studied extensively using the *G. mellonella* model [26–31,34,35]. In general, three main approaches have been employed to study interactions between *Lactobacillus* species and other bacteria: (1) co-inoculation with a combination of bacterial cells, (2) treatment with *Lactobacillus* cell-free supernatants, and (3) pre-exposure of the larvae to one species prior to the infection with another. This co-infection model was used to investigate the relationship between *Lactobacillus acidophilus*, *Lactobacillus gasseri*, and enterotoxigenic *Escherichia coli*. In the present study, two approaches were used to establish infection: simultaneous infection with both species and a prophylactic approach where *Lactobacillus* strains were injected 4 h prior to the infection with *E. coli* [26]. The present study reported that the co-infection strategy was ineffective in controlling the *E. coli* infection, whereas the prophylactic approach led to a significant increase in survival of larvae treated with *L. acidophilus* and *L. gasseri* against different strains of enterotoxigenic *E. coli*. However, in the co-infection *G. mellonella* model used by Dijokaite *et al.*, 2021 it was reported that the co-infection of *G. mellonella* with *L. gasseri* and *Nesseria gonorrhoeae* failed to reduce the larval mortality despite the *in vitro* studies suggesting that *Lactobacillus* is capable of inhibiting *N. gonorrhoeae* virulence mechanisms [27]. The prophylactic approach using inoculation of *G. mellonella* with *Lactobacillus kunkeei* prior to the infection with *P. aeruginosa* has also been shown to reduce the mortality in this model [27].

In addition to this, the *G. mellonella* burn wound model was used to study the relationship between *Lactobacillus spp* and *P. aeruginosa* in the context of wound infection using both the *Lactobacillus* cell-free supernatant treatment approach and the pre-inoculation of the wound bed with *Lactobacillus* cells prior to the infection with *P. aeruginosa* [29–31]. The burn wound model was also used to identify the probiotic effect of human commensal skin bacterium *Cutibacterium acnes* (formerly *Propionibacterium acnes*) against *P. aeruginosa*, suggesting a competitive relationship between these two bacteria (Figure 1). The model was also used in the present study to validate the proposed mechanism of action underpinning this competitive relationship by confirming that glucose was required for full virulence of *P. aeruginosa* and that *C. acnes* was capable of scavenging glucose from the wound microenvironment [32]. Several other studies explored the effect of the route of administration of prophylactic bacteria on pathogen virulence potential. In Menta *et al.* [33], it was reported that oral pre-treatment with cells of a probiotic *E. coli* strain increases *G. mellonella* survival when challenged with *Salmonella typhimurium*, whereas injection treatments decreased the survival [33]. In Grounta *et al.* [34], higher survival during *Listeria monocytogenes* challenge was observed in the larvae pre-infected with heat-killed *Lactobacillus* cells [34]. The relationship between *Enterococcus faecalis* and *Lactobacillus crispatus* was also investigated using the *G. mellonella* co-infection model, showcasing that *L. crispatus* eradicates *E. faecalis* cells in a contact-independent manner [35]. The capacity of *G. mellonella* models to support high-throughput screening and rapid data generation makes them particularly well suited for probiotic discovery and the subsequent molecular characterisation of interbacterial and interkingdom interactions.

The relationship between *P. aeruginosa* and *Streptococcus anginosus* was also investigated, revealing that co-infection with these species increases the mortality. The mechanism underpinning this effect was also explored, and it was shown that *S. anginosus* triggered a reversion of the mucoidal *P. aeruginosa* phenotype, leading to higher production of pyocyanin and enhanced virulence as a result [36]. Furthermore, the co-infection *G. mellonella* model has been used to study the infection and biofilm dynamics between the Cystic Fibrosis-derived isolate of *P. aeruginosa* and anginosus group streptococci. This revealed that these two species are capable of forming cooperative relationships leading to enhanced virulence factor production, such as pyocyanin and elastase, ultimately resulting in increased pathogenicity (Figure 1) [36,37]. The relationship between the opportunistic pathogens *Acinetobacter baumannii* and *Klebsiella pneumoniae* has been described as synergistic, where *A. baumannii* is capable of feeding on the by-products of sugar fermentation in *K. pneumoniae*, increasing the mortality in the *G. mellonella* co-infection model [17]. The breadth of interactions described with the help of the *G. mellonella* model and experimental design flexibility that this model affords uniquely positions *G. mellonella*, among other *in vivo* models, as an attractive step between *in vitro* studies and higher animal models.

## *Galleria* as a model to investigate host immune responses

**Figure 2:**
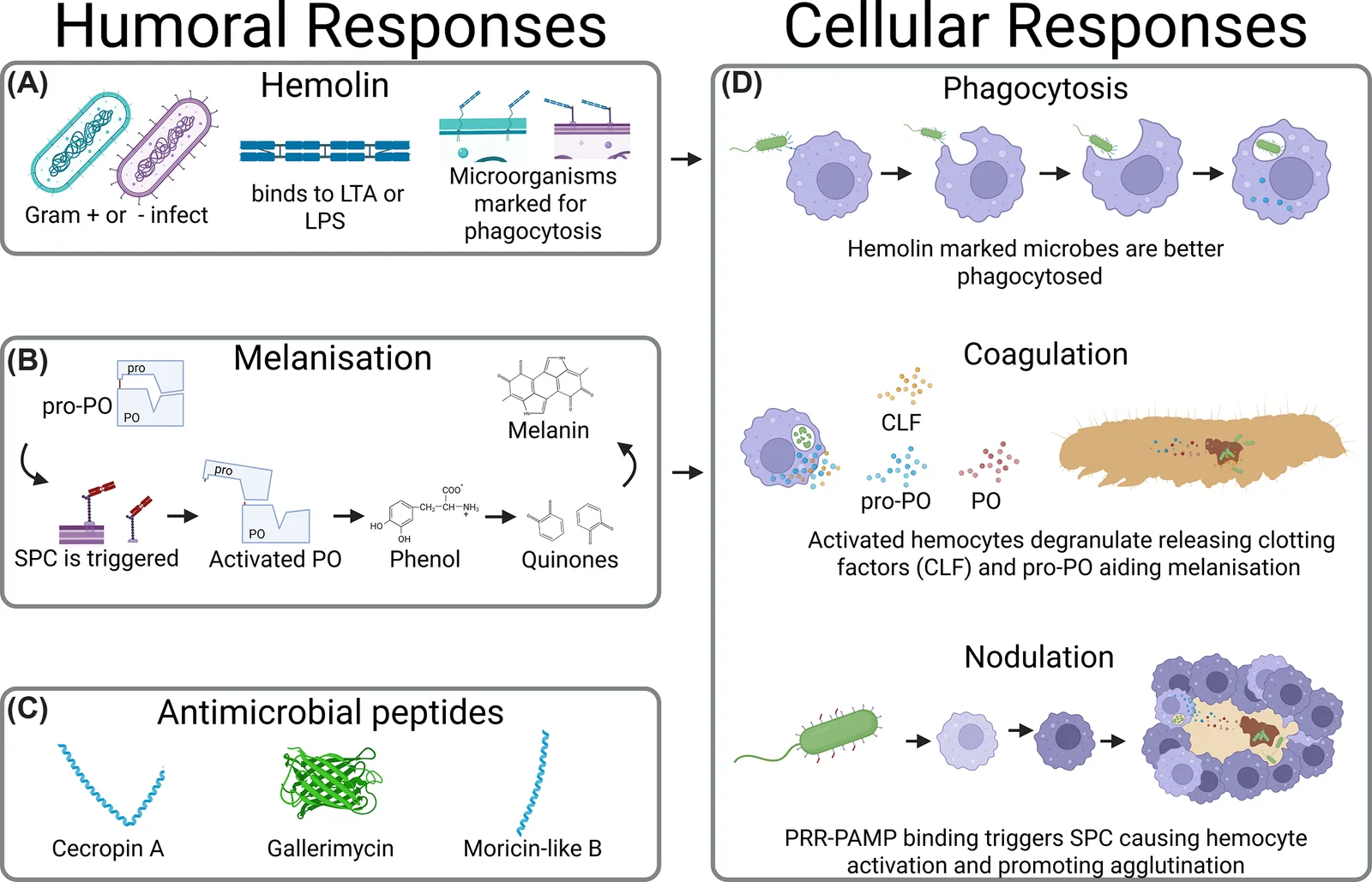
Diagram of *G. mellonella* immune system responses to microbial infection On the left, panels depict aspects of the humoral response to infection, including hemolin, melanin, and a selection of the several AMPs found in *G. mellonella* (panels (A), (B), and (C), respectively). On the right, panel D highlights the cellular responses to infection and how hemocytes facilitate phagocytosis, nodulation, and coagulation of bacteria. In panel (**A**), Gram-positive and Gram-negative bacteria are depicted. Additionally, the panel shows hemolin binding to lipoteichoic acid (LTA) and lipopolysaccharide (LPS) on the surface of each bacterium. Panel (**B**) depicts how the inactive pro-form of phenoloxidase (PO) is activated by the serine protease cascade (SPC) to facilitate melanin production. Panel (**C**) presents three of the many AMPs found in *G. mellonella*. Panel (**D**) depicts snapshots and mechanistic insight of how the *G. mellonella* immune system may respond to an infection, highlighting the role of hemocytes in the cellular response. This figure was created based on references [1,2,38–40] and was generated using BioRender.com.

Recently, the immune response of *G. mellonella* has been characterised in greater depth, improving our understanding of how it limits infection. The *G. mellonella* co-infection model enables assessment of how bacterial–bacterial interactions affect the host by evaluating the larval immune response utilizing a range of histological and transcriptomic approaches [21,25–27,30,39,38]. *G. mellonella* possesses a robust innate immune system with elements that closely resemble those of mammalian and human immune factors, both through function and structure [9,12,13]. The components of the *G. mellonella* immune system include lymph nodes, fatty bodies, and mesoderm-originating haemocytes (equivalent of innate immune cells) [1]. Its immune system is primarily distinguished into cellular and humoral responses. The cellular immune response of *G. mellonella* is primarily mediated via haemocytes. They can neutralize pathogens using phagocytosis, nodulation, coagulation mechanisms, and melanisation (Figure 2D) [40]. Humoral responses are based on soluble molecules that are secreted in response to pathogens, such as lytic enzymes, melanin, and AMPs, including gallerimycin, cecropin, and gloverin [41] (Figure 2). These AMPs are a key part of the larval immune system and are among the best described peptides in this invertebrate.

*P. aeruginosa* has been studied extensively with respect to how the larvae respond to infection. Transcriptomic analysis of the infected larvae revealed that several AMPs are induced by infection with *P. aeruginosa* including cecropin, gallerimycin, galiomycinproline-rich peptide 1 and 2, lebocin-like anionic peptide 1, moricin-like peptide B, and cecropin D-like peptide [42]. However, *P. aeruginosa* does mount a response to these AMPs, producing extracellular proteinases capable of degrading *G. mellonella* immune factors [43]. *G. mellonella* produces a large repertoire of AMPs, permitting targeted investigation of individual peptides based on the infecting pathogen, as AMP efficacy and roles vary during infection. These peptides are produced primarily in the fatty body of the larvae. Their mode of action is broad and ranges from suppressing intrabacterial processes to disrupting the microbial membrane [42]. The *G. mellonella* AMP cecropin A has also been reported for its biofilm-disrupting effect on *E. coli* biofilms, suggesting it may have potential as a novel antivirulence therapeutic [44]. *Mycobacterium tuberculosis* was shown to trigger an increase in the immunoglobulin protein hemolin (Figure 2A), β-actin, cecropins, and gloverin [45]. Furthermore, elevations in cecropin, galiomycin, gallerimycin, and alipophorin-III have also been detected in *G. mellonella* infections with *S. brasiliensis, E. coli*, *P. entomophilia*, and *C. albicans* [46–48]. These AMPs carry out a similar function to the AMPs in higher animals and humans. This highlights a conservation between higher-order model organisms and *Galleria* with respect to AMP induction.

Microbial toxins are a significant contributor to pathogenesis, and the immune system of *G. mellonella* possesses several mechanisms to counteract them, further enhancing the utility of this model for studying host–pathogen interactions. *G. mellonella* apolipoprotein activity, which is involved in the detoxification processes, has been shown to respond to exposure to the *P. aeruginosa*-derived virulence factor, exotoxin A, showcasing that this model's response not only to the infection with a live bacterium but also to isolated toxins [38]. Infection with *P. aeruginosa* has been shown to up-regulate zinc uptake systems in *G. mellonella*, which mimics the nutritional responses to infection in mammalian organisms [49]. Additionally, the immunomodulatory activity of *Lactobacillus spp* treatment against *P. aeruginosa* has been evaluated by measuring the mRNA expression levels of *gallerimycin* and *relish/NF-kB* in the *G. mellonella* burn wound model [30]. The present study reported not only a significant up-regulation of these genes in the *P. aeruginosa* infection, but also that *gallerimycin* was significantly down-regulated in the groups treated with *Lactobacillus spp*, whereas *relish/NF-kB* was not affected. Relish is an NF-κB–like transcription factor in the insect immune deficiency pathway, which is analogous to the NF-κB signaling pathway in higher animals and plays a central role in regulating early innate immune responses [30].

Histology has been widely used to investigate the impact of bacterial infection on the *G. mellonella* tissues and the dissemination of the pathogen in this model. This approach has been utilised to showcase how the pathogen impacts the tissues and disseminates throughout the larval body. Kordaczuk *et al*. used a histological approach to show how *Pseudomonas* damages the cuticle and epidermal layer and enters the haemocoel [48]. This histological approach also allows researchers to visualize infection dynamics, for example, the visualisation of melanisation and pathogen dissemination kinetics was recorded in the dual infection of *G. mellonella* with *C. albicans* and *S. aureus* and co-infection with *C. albicans* and *G. vaginalis* [21,22,25]. Visualisation of dual infections has also been used to showcase the proliferation of probiotic strains of *Lactobacillus spp* in the co-infection with enterotoxigenic *E. coli* [26]. Furthermore, histological analysis enables detailed characterisation of the larval host response by enabling the comparison of total lesion grade, the amount of melanised nodules, and hemocyte response [27]. Additionally, the larval immune response can be assessed through SDS-PAGE electrophoresis, and hemolymph proteins can be isolated and tested individually *in vitro* for antimicrobial activity, thereby broadening the opportunities for follow-up studies using this model [38,48].

## Bacteria-substrate interactions

**Figure 3:**
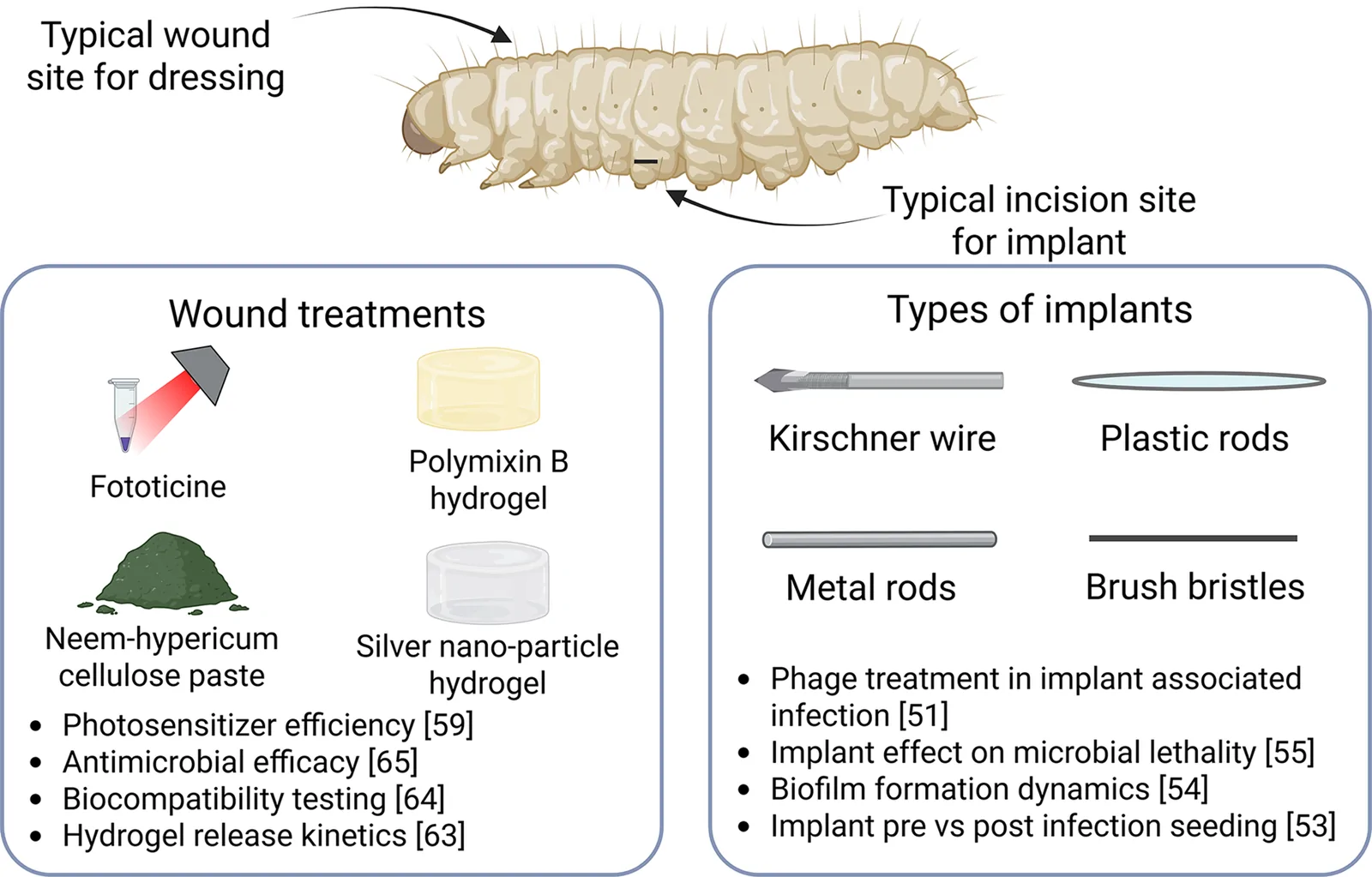
Schematic representation of bacteria–substrate interactions investigated within *G. mellonella* larvae At the top of the figure, the relevant site on the larva is indicated where substrates are applied as wound dressings or implants. The lower left shows wound dressings that have been validated within the *G. mellonella* model, such as hydrogels, antimicrobial pastes, and antimicrobial photodynamic therapy. The site of injury (e.g., burn wound) is also depicted. The lower right half illustrates the different substrates used as implants in the *G. mellonella* model. Additionally, examples of measured outcomes are provided for each substrate. This figure was created based on references [51,53–55,59,63–65], and was generated using BioRender.com.

Perhaps the most multifaceted application of the *G. mellonella* model to investigate bacterial interactions has been in the field of bacteria-substrate interactions. *G. mellonella* has been widely used as a drug efficacy assessment model, but recently it has also been developed as a bacteria-substrate model, such as a burn wound model to investigate topical treatments for bacterial infections, as well as multiple versions of the implant infection model [50–54] (Figure 3). In the context of the present review, an inert physical entity applied to the model is referred to as the substrate.

Medical-device-associated infections are a major focus of microbiological research due to the unique biological and physicochemical environments in which these infections develop. However, similarly to other models, studying foreign body-associated infections *in vivo* is a logistical, financial, and ethical challenge. The implant model using *G. mellonella* offers an alternative *in vivo* approach to study this type of infection. This model typically uses toothbrush bristles, K-wire or other metal (steel or titanium), or plastic implants that are inserted into the *G. mellonella* body [54–57]. There are two approaches to establishing the infection in this model: (1) insertion of a sterile implant and subsequent injection of the pathogen and (2) insertion of the implant pre-seeded with the pathogen and investigating the progression of the infection from there. The implant model allows for the evaluation of biofilm formation on the surface of the implant as well as the bioburden in the larval haemolymph, which provides a greater insight into infection progression dynamics [54]. This model has allowed the evaluation of the phage therapies in the medical device-associated methicillin-resistant *S. aureus* infection using the K-wire [52]. The plastic-containing medical devices and plastic-degrading pathogenic strains of *P. aeruginosa* have also been characterised using this model [56]. Additionally, as biofilm formation on medical devices is one of the major challenges with medical device-associated infections, this model has been shown to facilitate the investigation of *S. aureus*- and *E. faecalis*-implant-associated infections. This included bacterial attachment to the implant, the biofilm development differences between pre-seeded implant and implant-in-larvae approaches, and post-antibiotic treatments of the implant [50].

The *G. mellonella* burn wound model has been used to investigate the relationships between bacteria within the wound microenvironment and to evaluate the efficacy of various substrates as topical treatments and wound dressings for wound infections [30,31,58,59]. Despite *G. mellonella*'s comparatively simple skin anatomy and lack of a complex immune response, this model is capable of determining the efficacy of several antimicrobial substrates. It has been used to study antimicrobial photodynamic therapy and validate the efficacy of AMPs derived from Winter flounder (*Pleuronectes americanus*) against *A. baumannii* [60,61]. Furthermore, this model has been used to characterise the dose-dependent antibiotic effect of liquid topical cationic bolalipid and gentamicin application to *A. baumannii* burn wound infections [62]. Additionally, it has been used to validate novel approaches of antibiotic delivery to the burn site, such as peptide hydrogels loaded with polymyxin B and hydrogels loaded with silver nanoparticles and *Origanum vulgare* essential oil against *P. aeruginosa* [63,64]. The *G. mellonella* burn wound model was used to showcase the efficacy and biofilm-detaching properties of a neem-hypericum cellulose-based paste and liquid solution on *P. aeruginosa* and *S. aureus*, leading to a significant increase in *G. mellonella* survival [65]. The antibiotic effect of hemithioindigo compounds against MRSA infection has also been characterised in this model [59]. A topical liquid treatment of antimicrobial scorpion-derived peptide has been shown to be effective against *A. baumannii* infection in the *G. mellonella* burn wound model [66]. In the context of the current antimicrobial resistance crisis, *G. mellonella* is a useful tool capable of providing insights and screening for novel therapeutic agents and strategies.

Overall, the *G. mellonella* burn wound model is well suited to both high-throughput screening and a broad range of applications, enabling dose-dependent treatment assays and the evaluation of diverse substrate formulations—including liquids, pastes, and hydrogels—as well as a wide variety of active compounds applied to wound infections. This makes this model a valuable tool in the discovery of novel therapeutic solutions to treat infections in the antibiotic resistance crisis.

## Limitations

Despite the numerous advantages of the *G. mellonella*-based model, it undoubtedly has a number of limitations that should be considered when planning research using this model. To begin with, if the larvae are procured from a commercial source such as pet or fishing shops, it may be hard to control the larval quality and age. However, this can be rectified by breeding larvae in-house or purchasing larvae from a research facility where the larvae have been inbred and sequenced to ensure the high quality of the larvae. The other limitation of this model is the variability of the required inoculum for infection establishment between pathogens. The infection-triggering inoculum differs between different strains of the same species as well; thus, the correct inoculum needs to be confirmed for each strain in this model to optimise studying pathogen interactions. In addition to that, *G. mellonella* does not possess complex organ systems or a closed-circulation system and adaptive immune system, which makes it sub-optimal to fully investigate many pathogens *in vivo*.

## Conclusion

*Galleria mellonella* overcome several of the hurdles that researchers face in *in vivo* research, such as affordability, ethical considerations, and regulations. The emergence of new *G. mellonella*-based models has reinforced its utility as both a replacement system and a versatile tool for investigating bacterial infections *in vivo*, with each new application expanding its experimental scope.

## Summary

- The *G. mellonella* research model is free from ethical restrictions, which enables the design of well-controlled, high-power, and multi-parameter bacterial interaction studies.
- The model has been used extensively to characterise interbacterial interactions, interkingdom interactions, interactions with the host immune system, and interactions with various substrates such as wound dressings and medical implants.
- Interactions can be tracked in real time, and the molecular mechanisms underpinning these interactions can be interrogated using the *G. mellonella* model.

## Open Access

Open access for this article was enabled by the participation of the University of Southampton in an all-inclusive Read & Publish agreement with Portland Press and the Biochemical Society under an agreement with JISC.

## Abbreviations

AMPs: Antimicrobial peptides
CFU: Colony forming units
CLF: Clotting factors
LTA: Lipoteichoic acid
LPS: Lipopolysaccharide
PO: Phenoloxidase
PRR-PAMP: Pattern recognition receptors-pathogen-associated molecular patterns
SPC: Cerine protease cascade
